# Fermentation Conditions and Media Optimization for Isocitric Acid Production from Ethanol by* Yarrowia lipolytica*

**DOI:** 10.1155/2018/2543210

**Published:** 2018-02-07

**Authors:** Svetlana V. Kamzolova, Roman V. Shamin, Nadezda N. Stepanova, Grigorii I. Morgunov, Julia N. Lunina, Ramil K. Allayarov, Vladimir A. Samoilenko, Igor G. Morgunov

**Affiliations:** ^1^G.K. Skryabin Institute of Biochemistry and Physiology of Microorganisms, Russian Academy of Sciences, Pr-t Nauki 5, P.O. Box 142290, Pushchino, Moscow, Russia; ^2^Peoples' Friendship University of Russia (RUDN University), Miklukho-Maklaya Str. 6, P.O. Box 117198, Moscow, Russia; ^3^Moscow Technological University (MIREA), Vernadskogo Pr. 78, P.O. Box 119454, Moscow, Russia

## Abstract

Isocitric acid exists in the form of four stereoisomers, of which only the* threo*-Ds-form (ICA) is a natural active compound, an intermediate of Krebs cycle, and suitable for nutritional and pharmaceutical use. In this paper, we propose a method for ICA production from ethanol by yeast* Yarrowia lipolytica*. The effects of temperature, pH of the medium, and aeration on the growth of the producer* Y. lipolytica* VKM Y-2373 and synthesis of ICA were studied. An optimal fermentation regime, which ensures a good growth of the producer and directed synthesis of the target product, was determined. The producer is advised to carry out cultivation at 29°C and various pH of the medium and the oxygen concentration (pH 5 and pO_2_ 20–25% (of saturation) during the growth period and pH 6 and pO_2_ 50–55% (of saturation) during the acid formation) on a nutrient medium containing an increased content of zinc (0.6 mg/L), iron (1.2 mg/L), and 30 mM itaconic acid (inhibitor of isocitrate lyase—the key enzyme of ICA metabolism) should also be introduced into the nutrition medium. Such fermentation production mode provides 90.5 g/L ICA with process selectivity of 80%, mass yield (*Y*_ICA_) of 0.77 g/g, and energy yield (*η*_ICA_) of 0.278 g/g.

## 1. Introduction

It is well known that the target of hypoxia is aerobic energy metabolism, occurring in mitochondria [1–8]. The authors of the mentioned article reported that during hypoxia the supply of oxygen is greatly disturbed, and the hypoxic conditions of various disease severities are developed. As a consequence, the protective mechanisms (stress reactions), mobilizing the energy resources and structural body, and activating the circulatory and respiratory functions are included in humans. The operation of such reactions is associated with the sharp increase in energy consumption. Excessive or prolonged stress reactions are accompanied by a progressive depletion of the body, which in turn leads to pathological changes of internal organs and tissues. In these circumstances, the development of pharmaceutical agents, (1) to increase the body's resistance to the influence of stress and hypoxic factors, (2) to maximize the use of oxygen supply in the body, (3) to increase the production of energy substrates, and (4) to protect the internal organs from pathological changes, becomes relevant. We suggested that the Krebs cycle intermediate, isocitric acid, can contribute to the normalization of energy metabolism, the adaptation to adverse environmental conditions, to stimulating physical performance, to preventing the development of fatigue, and to protecting the internal organs and tissues from hypoxic factors. Isocitric acid is the only metabolite of the Krebs cycle that can unblock succinate dehydrogenase and, hence, promote cell respiration even at durable and intense stresses [9]. In this case, cell respiration is probably not increased. Presumably, isocitric acid merely promotes the utilization of oxygen available in cells.

The bottleneck in the development and practical application of this simple, nontoxic, and moderately priced preparation is the absence of industrial production of isocitric acid of required quality, which is different from the products of chemical synthesis by natural isomeric composition. It is known that the presence of even small impurities in the chemically produced preparations decreases the quality of preparations and makes them toxic for plants, animals, and humans [10–12]. It should be noted that isocitric acid has four isomers: erythro-Ds, erythro-Ls, threo-Ls, and threo-Ds. Among these isomers, only threo-Ds-isocitric acid (ICA) is a natural compound, which is present in every living cell [13] and, hence, only this isomer is of practical interest for food and medicine. ICA sources include the leaves and stems of some plants, fruits, and berries, especially in* Hylotelephium spectabile* (syn.* Sedum spectabile*); however, the ICA content of these substrates is so low that they cannot satisfy increased need in this acid.

Microbiological production of ICA could be a promising alternative to chemical synthesis and recovery from plants. ICA is produced by* Yarrowia lipolytica* yeast when its growth is limited by biogenic elements, in particular, nitrogen source [14–22]. In these processes, *n*-alcanes and plant oils are usually used as carbon substrates, and* Y. lipolytica* produces ICA and byproduct, citric acid (CA), in approximately equal amounts. It should be emphasized that* Y. lipolytica* yeasts as well as ICA are generally recognized as safe (GRAS) [12, 20].

At present, the ethanol is considered to be a promising carbon source in various biotechnological processes, because it can be produced from sugarcane, beet, corn, lignocellulose, and other renewable materials. Ethanol as a substrate for growth has several advantages over other substrates. It does not contain harmful impurities; it is well assimilated by yeast and dissolves in water in any proportions. Since ethanol is used in the human diet, the products derived from it do not require additional purification from the residual substrate. Several companies in the US and Switzerland have created food products based on microbial biomass produced from ethanol [24, 25]. It should be noted that the large-scale production of ICA from ethanol is still limited by the lack of basic knowledge about fermentation conditions conducive to product overproduction.

The aim of this work was to study the effects of fermentation parameters (temperature, pH, and aeration) and components of nutrition medium (zinc, iron, and itaconic acid) on the growth and ICA synthesis by* Y. lipolytica* grown on ethanol.

## 2. Materials and Methods

### 2.1. Chemicals

All chemicals were of analytical grade (Mosreactiv, Russia). Ethanol was purchased from “Kupavnareaktiv” (Russia).

### 2.2. Microorganism Used

Experiments were carried out with the yeast strain* Y. lipolytica* VKM Y-2373, which is able to produce ICA in marked amounts [19]. The strain was maintained at +4°С on agar with paraffin.

### 2.3. The Determination of Cultivation Conditions

To determine the effect of temperature, pH, and aeration on growth of* Y. lipolytica* VKM Y-2373, the yeast was cultivated in an ANKUM-2M fermenter with a 1.5-l working volume of the medium containing (g/L) (NH_4_)_2_SO_4_, 0.126; K_2_HPO_4_, 0.875; МgSO4·_7_H_2_O, 1.5; Ca(NO_3_)_2_·4H_2_O, 0.136; NaCl, 0.5; double Burkholder's trace element solution; yeast autolysate, 8 ml/L. For preparation of yeast autolysate 10 kg of the commercial press baker's yeast (Saf-Neva, Ltd., Russia) was suspended in 6 L of distilled water. Autolysis was performed in the presence of 250 microliters of ethanol and 80 microliters of chloroform at 50°C during 24 h. Then sample was centrifuged at 11000*g* for 20 min at 4°C in order to remove cell debris. Amino nitrogen content in yeast autolysate consisted of 0.45%. Ethanol was added by portions (2 g/L) up to 10 g/L. The temperature, pH, and the concentration of dissolved oxygen in the medium were maintained at a specified level as described in the Results and Discussion.

To determine the effect of fermentation parameters on ICA production, the yeast was cultivated in a 10 L ANKUM-2M fermenter (Russia) with 5 L of the medium (initial volume) containing (g/L) (NH_4_)_2_SO_4_, 3.0; KH_2_PO_4_, 2.0; K_2_HPO_4_, 0.2; МgSO_4_·7H_2_O, 1.4; Ca(NO_3_)_2_·4H_2_O, 0.8; NaCl, 0.5; Burkholder's trace element solution; yeast autolysate, 8 ml/L. Pulsed additions of ethanol (by 2–6 g/L) were performed as the pO_2_ value changed by 10% indicating a decrease in respiratory activity of cells due to the total consumption of the carbon source. During growth phase (up to 24 h), the fermentation conditions were maintained automatically at the constant level: temperature 29 ± 0.5°C; pH of 5 ± 0.1, pO_2_ of 20–25% (of saturation); agitation 800 rpm. During acid formation phase (after 24 h), pH and pO_2_ values in the medium were maintained as described in the Results and Discussion. Cultivation was carried out for 6 days.

To determine the optimal concentrations of zinc and iron ions for ICA production, the strain was grown in a fermenter under nitrogen limitation to the phase of active acid formation (biomass of 10-11 g/L). Cells were separated from the culture liquid by centrifugation, washed with 0.9% NaCl, and suspended in 50 mM phosphate buffer (pH 7.0). The cell suspension was placed in 750-ml Erlenmeyer flasks with 50 ml of the medium, which was free of nitrogen, microelements, and vitamins but contained ethanol (up to 10 g/L) and incubated on a shaker (180–200 rpm) at 29°С for 36 h. By the end of the experiment, pH of the medium slightly decreased (by 0.4–0.5 units), biomass remained at a constant level of 2.5 g/L, and the cell viability remained unchanged. The determination of the number of viable cells was carried out by inoculating on agar nutrient media with glucose as a carbon source.

To determine the optimal concentration of itaconic acid, the strain was grown at 29°C and various pH and pO_2_ values (pH 5 and pO_2_ of 20–25% during the growth period and pH 6 and pO_2_ of 50–55% during the acid formation period) on a nutrient medium containing increased Zn^2+^ (0.6 mg/L) and Fe^2+^ (1.2 mg/L).

### 2.4. Calculation of Fermentation Parameters

In pH-auxostat, the maximum specific growth rate (*μ*_max_) is equal to the dilution rate *D*.

Methods for calculation of mass yield coefficient of ICA production (*Y*_ICA_) and the specific rate of ICA production (*q*_ICA_) were described earlier [19].

### 2.5. Measurement Techniques

Methods of analysis of nitrogen, biomass, ICA, and CA were described earlier [19].

## 3. Results and Discussion

### 3.1. Effect of Fermentation Parameters on the Growth and ICA Production

**Figure 1:**
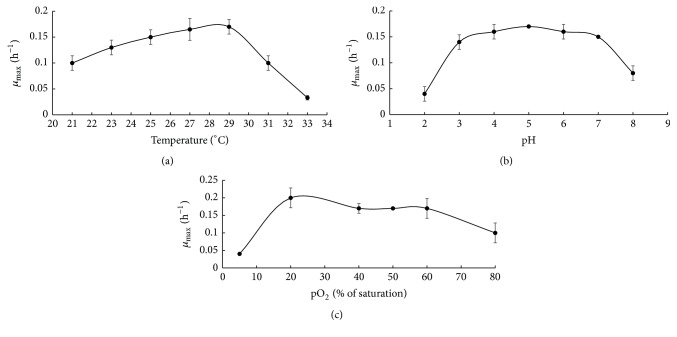
The effect of fermentation parameters ((a) temperature, (b) pH medium, and (c) pO_2_ (of saturation) on the maximal specific growth rate (*μ*_max_)).

In order to select the optimal conditions for growth of* Y. lipolytica* VKM Y-2373, the effect of temperature, pH, and aeration was studied under conditions of pH-auxostat. Method of pH-auxostat is a variant of the method of continuous cultivation of microorganisms, in which medium serves as titrant and is added to a fermenter according to a signal of pH-meter, when pH of the culture liquid in a fermenter was changed from the established value. Under steady-state regime, all components of the medium in a fermenter are in excess, and microbial cells grow practically at the maximal specific growth rate (*μ*_max_), which is equal to the dilution rate of the medium. This method made it possible to study the effect of physical and chemical factors on *μ*_max_ [26]. The data on the effect of temperature, pH, and aeration on *μ*_max_ are given in Figure 1.

In experiments on the effect of temperature, the strain was grown at pH of 5. The temperature was first decreased from 27 to 25, 23, and 21°C, elevated to the initial level (27°C), and then raised to 29, 31, and 33°C. As seen from Figure 1, the optimal temperature for yeast growth was 29°C (*μ*_max_ was 0.17 h^−1^). When the temperature was lowered to 21°C, the *μ*_max_ value gradually decreased to 0.1 h^−1^. Elevation of the temperature from 29 to 33°C caused a decrease in the yeast growth 5.7 times.

The effect of pH on the *μ*_max_ was studied at 29°C; the concentration of dissolved oxygen was maintained at the constant level (pO_2_ = 60% of saturation). As seen in Figure 1, the optimal pH medium for yeast growth was 5.0 (*μ*_max_ was 0.17 h^−1^). Over the pH range from 7 to 3, the *μ*_max_ remained at a high level (0.14–0.15 h^−1^). When pH dropped below 3, yeast growth was strongly inhibited, and, at pH 2, the *μ*_max_ value was as low as 0.04 h^−1^. When pH raised to 8, yeast growth was also inhibited, and, at pH 8, the *μ*_max_ value was 0.08 h^−1^.

The effect of oxygen concentration on the *μ*_max_ value in the range from 5 to 80% (of saturation) was studied at 29°C and pH 5. As seen in Figure 1, the optimal oxygen concentration for yeast growth was 20% (of saturation) (*μ*_max_ was 0.20 h^−1^). Over the pO_2_ range from 40 to 60% (of saturation), the *μ*_max_ remained as a high level (0.17–0.18 h^−1^). The *μ*_max_ value decreased by 2 times at oxygen concentration of 80% (of saturation). The extremely low aeration (5% (of saturation)) limited growth (*μ*_max_ was 0.04 h^−1^) and induced the formation of pseudomycelial forms instead of rounded single cells observed at oxygen concentration of 20–60% (of saturation).

In order to select the optimal fermentation conditions for ICA production of* Y. lipolytica* VKM Y-2373, the effect of pH and aeration was studied under conditions of nitrogen limitation of growth in fed-batch mode. Ethanol creates certain difficulties during cultivation of microorganisms due to its toxicity. High concentrations of ethanol inhibit the cell growth and the transport of various nutrients and alter the permeability of the cytoplasmic membrane [27]. In this connection, the initial concentration of ethanol in the medium was 2 g/L. When pO_2_ in the medium exceeded its normal level by 10% (this increase in the concentration of dissolved oxygen in the medium indicated a reduction in the respiratory activity of yeast cells due to ethanol consumption), ethanol was replenished by the addition of a new portion of this nutrient in an amount corresponding to its concentration of 2–6 g/L in the cultivation medium. The obtained data are shown in Table 1.

The effect of pH in the range from 3 to 7 on ICA production from ethanol was studied at 29°C and dissolved oxygen concentration (pO_2_) of 50% (of saturation). As seen from Table 1, the maximum ICA production (59.0 g/L) was observed at pH 6; in this case, a ratio between ICA and CA amounted to 1.9 : 1, and product yield (*Y*_ICA_) achieved 0.55 g/g. When pH was lowered to 3, ICA production gradually decreased to 13.75 g/L. Elevation of pH from 6 to 7 caused a decrease in ICA production 4.8 times. It should be noted that at pH 3, pH 4, and pH 4.5* Y. lipolytica *VKM Y-2373 produced equal amounts of ICA and CA.

The effect of pO_2_ on ICA production in the range from 5–10 to 75–80% was studied at 29°C and pH 6.0. As seen from Table 1, the maximum ICA production (59.0 g/L) was 50–55% (of saturation); in this case, a ratio between ICA and CA amounted to 2 : 1, and product yield (*Y*_ICA_) achieved 0.55 g/g. The intensive ICA production was also observed at pO_2_ 30–35, 40–45%, and 60–65% (of saturation) and decreased by 1.7 times at oxygen concentrations 20–25 and 70–80% (of saturation). The extremely low aeration (5–10%) inhibited ICA production.

Therefore, it can be recommended to maintain pH and pO_2_ at the levels of 5.0 and 20% (of saturation), respectively, in the exponential phase for optimal culture growth and then to adjust pH to 6.0 and pO_2_ to 50–55% (of saturation) in the stationary phase for active ICA production by* Y. lipolytica* VKM Y-2373.

Literature data on the effect of temperature on physiology of* Y. lipolytica* are scarce. It is noted that temperature must be very well defined and optimized depending on the strain and the growth phase of producer. It was reported that* Y. lipolytica* grow well at temperatures between 20 and 37°C [28]. Moeller et al. (2007) found that, during* Y. lipolytica* growth, the temperature optimum was in the range of 30–34°C (*μ*_max_ = 0.132 h^−1^), but the highest concentration of CA was obtained at 30°C (41 g/L CA and product yield (*Y*_CA_) of 0.55 g/g) [29]. Anastassiadis et al. (2002) reported the temperature optimum for CA production to be 30-31°C for* Candida oleophila* [30]. The authors of the last article explain this effect by stating that temperature influences regulation and transport systems of metabolites. The concentration of intracellular ICA decreased sharply with raising temperature and consequently the velocity of active transport system in* C. oleophila*, while intracellular CA concentration remained approximately constant; a higher intracellular CA/ICA ratio is to be recorded at higher temperatures, in contrary to an almost identical extracellular ratio.

It is known from the literature that variation of oxygen within the physiological range for* Y. lipolytica* yeast significantly influences the growth and acid formation. As noted,* Y. lipolytica* is an obligatory aerobe and does not grow without oxygen [11, 12]. The dissolved oxygen concentration is considered as the major factor affecting yeast morphology. Specifically, when growth occurred at low or zero pO_2_ the mycelial and/or pseudomycelial forms predominated over the yeast form independently of the carbon and nitrogen sources used [31]. For* Y. lipolytica*, grown on fatty materials, the good growth and the CA and ICA production were observed at an elevated level of dissolved oxygen, while the dissolved oxygen level close to zero led to the accumulation of intracellular lipids and extracellular lipase [32, 33]. For* Y. lipolytica*, cultivated on waste glycerol-based media under conditions of limitation of growth by nitrogen, with a high level of pO_2_, the metabolism shifted toward the formation of polyols, while at a low pO_2_ level, the nitrogen deficiency led to CA production [34].

Literature data on the effect of pH medium on physiology of* Y. lipolytica* are scarce. Moeller et al. (2007) found that the highest value of *μ*_max_ (0.192 h^−1^) was at pH 6.5, whereas the largest amount of CA (24.91 g/L), the highest selectivity of the process (89.9% CA), and the maximum yield (*Y*_CA_) (0.22 g/g) were obtained at pH 6.0 [29]. It was reported that* Y. lipolytica* grows perfectly well over a wide pH range, but pH has a decisive influence on the metabolite pattern accumulated by this yeast. It was reported that* Y. lipolytica* is able to shift its metabolite pattern from polyol production at pH 3.5 toward CA and ICA production at pH 5.5 [35, 36]. Recently we found that ICA production from rapeseed oil and the ratio between ICA and CA depended on pH of the medium:* Y. lipolytica* produced predominantly ICA at pH 6.0, while equal amounts of ICA and CA were accumulated at pH 4.5 [19]. We suggested that pH showed no direct effect on mechanism of ICA synthesis but influenced considerably the permeability of cell membranes to both substrate and products.

### 3.2. Effects of Zinc and Iron

Earlier, we reported that zinc and iron play a key role in metabolism of* Y. lipolytica*. In particular, zinc limitation resulted in imbalance between the enzyme systems involved in primary ethanol oxidation, namely, alcohol dehydrogenase (EC 1.1.1.1) and aldehyde dehydrogenase (EC 1.2.1.3), which seemed to impose the accumulation of acetaldehyde, considered to be toxic for all cell functions [37, 38]. A relationship was found between the amount of Fe^2+^ and ICA production in* Y. lipolytica *grown on rapeseed oil, and, hence, we were successful in controlling the ICA/CA ration through the regulation of the iron-dependent enzyme aconitate hydratase (EC 4.2.1.1) by changing iron concentration in the medium [19].

**Figure 2:**
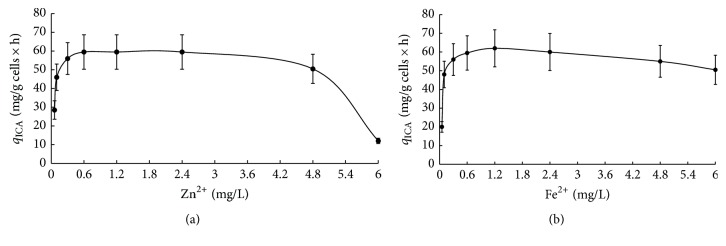
The effect of microelements ((a) zinc, (b) iron) on the specific rate of ICA production (*q*_ICA_).

In this study, the effects of zinc and iron concentrations on specific rate of ICA production (expressed in mg/g cells × h) of the producer* Y. lipolytica* VKM Y-2373 were studied within the ranges 0.06–6 mg/L and 0.05–6 mg/L, respectively. As seen from Figure 2, at low zinc level of 0.06 mg/L ICA synthesis was insignificant. An increase in zinc from 0.06 to 0.6 mg/L caused an increase in ICA production 2 times, and the high specific rate of ICA production was maintained within the range 0.6–4.8 mg/L. A further increase in zinc up to 6 mg/L imposed a sharp decrease in ICA production.

The effect of iron concentration (Fe^2+^) on ICA production was studied at Zn^2+^ of 0.6 mg/L. As seen from Figure 2, Fe^2+^ of 0.05 mg/L limited ICA production. An increase in Fe^2+^ from 0.05 to 1.2 mg/L increased ICA 3.1 times, and a further increase in zinc up to 6 mg/L does not lead to suppression of ICA synthesis.

Thus, the highest ICA production by* Y. lipolytica* VKM Y-2373 grown on ethanol occurred at high concentrations of Zn^2+^ (0.6 mg/L) and Fe^2+^ (1.2 mg/L).

### 3.3. The Effect of Itaconic Acid on ICA Production

In the next experiments, we investigated the effect of itaconic acid on ICA production from ethanol by* Y. lipolytica *at high concentrations of Zn^2+^ (0.6 mg/L) and Fe^2+^ (1.2 mg/L). The itaconic acid was used at concentrations from 7.5 to 40 mM; it was added in a fermenter in the phase of growth retardation and beginning the accumulation of acids (24 h of cultivation). Data on the effect of itaconic acid on the ICA production by* Y. lipolytica* VKM Y-2373 are shown in Table 2. In control experiments, the yeast was cultivated without inhibitor.

As seen from Table 2, the addition of itaconic acid up to 30 mM resulted in increase in ICA production from 64.3 to 72.19 g/L. Moreover, itaconic acid displaced the ratio of produced ICA to CA toward ICA. The maximum effect of itaconic acid was observed at its concentration of 30 mM (ICA/CA = 4.1 : 1 as compared to 2.7 : 1 in the absence of the inhibitor). Thus, the addition of itaconic acid resulted in a 100% increase in ICA/CA ratio toward the ICA production. The product yield (*Y*_ICA_) achieved 0.77 g/g at 20 and 30 mM itaconic acid. At 40 mM itaconic acid, ICA production and yield (*Y*_ICA_) were slightly decreased.

Earlier, the same correlation between itaconic acid and ICA production had been revealed when strain* Y. lipolytica* VKM Y-2373 was grown in the medium with rapeseed oil [22] and *n*-alkanes [39]. This phenomenon can be due to itaconic acid inhibition of isocitrate lyase (EC 4.1.3.1) (ICL), a key enzyme involved in the metabolism of ICA. Numerous studies confirmed that yeast ICL is a constitutive enzyme subject to catabolite repression. It is induced when the yeast grows on acetate, *n*-alkanes, ethanol, and fatty acids [40–43]. At the same time, glucose in the cultivation medium suppresses ICL [41, 42]. If the medium contains a mixture of hexadecane and glucose, ICL is suppressed until glucose is exhausted and then is induced providing the consumption of hexadecane [42]. The genetic studies of* Saccharomyces cerevisiae *showed that glucose suppresses ICL with the aid of ten amino acids inside the polypeptide chain with the involvement of the cAMP-dependent protein kinase [44]. The promoter region necessary for the activation of ICL synthesis is common to the genes encoding the enzymes involved in gluconeogenesis [45]. The deletion of the* ICL1* gene from the genome of* Y. lipolytica* not only inhibits the assimilation of acetate, ethanol, and fatty acids but also reduces the rate of growth of this yeast on glucose [46]. The data available in the literature for yeasts show that 2-phosphoenolpyruvate (PEP), 6-phosphogluconate, glucose 6-phosphate, pyruvate, and oxaloacetate are strong inhibitors of yeast ICL [47]. ICL is also inhibited by malate, fumarate, *α*-ketoglutarate, and citrate [48]. It should be noted that all the aforementioned inhibitors are intermediates of the major metabolic pathways and, hence, cannot be considered as specific inhibitors of ICL in growing yeast cultures. The inhibitory action of itaconic acid (a structural analogue of succinate) is also well known [22, 39, 49]. Experiments with* Y. lipolytica* showed that itaconic acid specifically inhibits ICL and is not metabolized by the cells [22, 39]. We reported that the addition of itaconic acid to the medium markedly inhibits ICL and, hence, reduces the amount of ICA cleaved by this enzyme [22]. This fact may well explain the displacement of ICA and CA formation toward the preferential synthesis of ICA, observed in the present study.

### 3.4. An Optimized Process of ICA Production

The optimized process of ICA production was carried out in a 10 L fermenter with a working volume of 5 L under optimal fermentation conditions (pH 5 and pO_2_ 20–25% (of saturation) during the growth and pH 6 and pO_2_ 50–55% (of saturation) during acid formation) in a balanced nutrition medium (Zn^2+^ 0.6 mg/L, iron 1.2 mg/L, and itaconic acid 30 mM).

**Figure 3:**
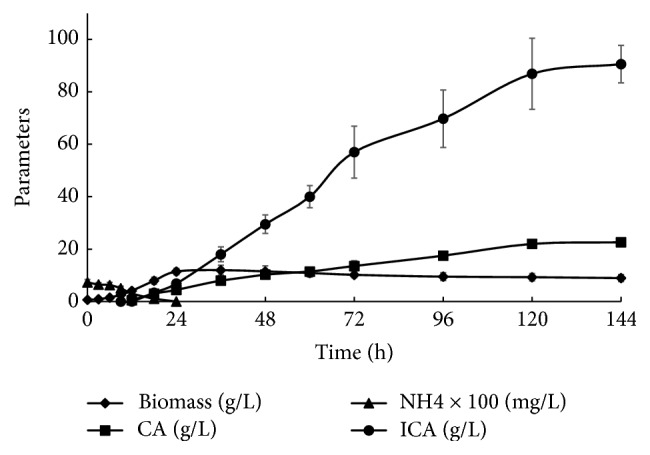
ICA production from ethanol of* Y. lipolytica* VKM Y-2373.

Figure 3 shows the curves of growth of* Y. lipolytica* VKM Y-2373, nitrogen consumption, ICA production, and accumulation of byproduct of fermentation, CA. During the exponential growth phase (up to 6 h), ICA did not accumulate. Its accumulation began in the growth retardation phase (12–36 h) and increased in the stationary phase (from 36 h) caused by depletion of nitrogen in the medium. After 144 h, the concentration of ICA in the medium reached 90.5 g/L and 22.6 g/L CA. The ratio of ICA/CA consisted of 4 : 1; therefore, the selectivity of fermentation was 80%. The mass yield of ICA (*Y*_ICA_) with consideration for medium dilution caused by the addition of KOH solution for pH stabilization during fermentation was calculated to be 0.77 g/g consumed ethanol. The productivity from ethanol consisted of 1.15 g/l·h.

The processes of ICA production from various substrates by yeast* Y. lipolytica* are presented in Table 3. In experiments with mutant* Y. lipolytica* NMM-149 grown on *n*-alkanes the ICA concentration reached 85 g/L with a yield of 1.23 g/g and a ratio between ICA and CA of 5.3 : 1 [14]. ICA production has been reported for wild strain of* Y. lipolytica* growing on ethanol: concentration reached 66 g/L with a yield of 0.66 g/g and a ratio between ICA and CA of 2.1 : 1 [23]. Heretsch et al. (2008) obtained ICA concentration of 93 g/L and CA concentration of 82.8 g/L, which are equivalent to a 1.1 : 1 ratio of ICA : CA and a yield of 0.65 g/g [16]. We found that wild strain* Y. lipolytica* VKM Y-2373, grown on rapeseed oil in the presence of itaconic acid, produced 70.6 g/L ICA with a yield of 0.82 g/g and a ratio between ICA and CA of 3.2 : 1, and the mutant strain* Y. lipolytica* 704-UV4-A/NG50 grown on rapeseed oil produced 86 g/L ICA with a yield of 0.95 g/g and a ratio between ICA and CA of 4.3 : 1 [19]. The cultivation of another mutant* Y. lipolytica* UV/NG in a pilot industrial fermenter in the presence of itaconic acid resulted in the production of 88.7 g/L ICA with a yield of 0.90 g/g and only 15.1 g/L of CA, so that the ICA : CA ratio was 6 : 1 [21]. It should be noted that the productivity of* Y. lipolytica* grown on rapeseed oil varied from 0.797 to 0.970 g/l·h [19] while the productivity of ethanol obtained in this study was higher (1.15 g/l·h).

### 3.5. Calculation of Energy Yields of ICA (*η*ICA)

Since carbon substrates possessed different energy capacities, it is inappropriate to compare the ICA mass yields from different substrates. It is more correct to compare the energy yields of ICA (*η*_ICA_) on different carbon sources.

We calculated the value of *η*_ICA_ which estimates a fraction of energy content of the substrate, incorporated into ICA, on the basis of mass and energy balance theory [50, 51]. Quantities that characterize mass and energy balance of cell metabolism are based on the generalized unit of reductivity, “redoxon”; it means an electron that can be transferred to oxygen; a former variant of this unit was an available electron. By definition, *η*_ICA_ of the product is a fraction of the total amount of substrate redoxons (available electrons), which is incorporated into the product.

The value of *η*_ICA_ was calculated using elementary composition of ICA and ethanol:

(1) $$\begin{array}{c} \eta_{ICA}=\frac{\left(\gamma_{ICA}\cdot \delta_{ICA}\right)}{\left(\gamma_{S}\cdot \delta_{S}\right)}\cdot Y_{ICA}, \end{array}$$

where *δ*_S_ and *δ*_ICA_ are mass fractions of carbon in ethanol (S) and ICA, respectively; *γ*_S_ and *γ*_ICA_ are reductance degree and the number of redoxons per 1 carbon atom of ethanol (S) and ICA, respectively.

For the substance (individual compound or a mixture) having the elementary composition of CH_p_O_n_N_q_ the reductance degree (*γ*) was calculated as follows:

(2) $$\begin{array}{c} \gamma =4+p-2n-3q, \end{array}$$

where 4 and *p* are the numbers of redoxons of the carbon and hydrogen atoms, respectively; *n* and *q* are the numbers of redoxons that lost their energy when they were bound up with the oxygen and nitrogen atoms, respectively. The elementary composition of ICA is C_6_H_8_O_7_ or CH_8/6_O_7/6_ after the calculation per 1 carbon atom, from which the reductance degree (*γ*_ICA_) is 4  +  8/6  −  2 · 7/6 = 3. The mass fraction of carbon in the molecule of ICA (*δ*_ICA_) is 0.375. Therefore, the value of *γ*_ICA_  ·  *δ*_ICA_ is 1.125. Correspondingly, the value of *γ*_S_  ·  *δ*_S_ for ethanol is 3.12. Thus, the value of *η*_ICA_ from ethanol can be calculated as (1.125/3.12) · *Y*_ICA_. Therefore, the value of *η*_ICA_* Y. lipolytica *VKM Y-2373, grown on ethanol, was 0.278 g/g.

In comparison, *η*_ICA_ for* Y. lipolytica *704-UV4-A/NG50, grown on rapeseed oil, was calculated on the basis of the value of *Y*_ICA_ of 0.95 g/g [19]. Taking into account the fact that the value of *γ*_ICA_  ·  *δ*_ICA_ comprised 1.125 and the value of *γ*_S_  ·  *δ*_S_ for rapeseed oil, which contained mainly oleic acid, averaged to 4.28, the value of *η*_ICA_ for rapeseed oil-grown* Y. lipolytica *704-UV4-A/NG50 was calculated as (1.125/4.28) · 0.95 = 0.250 g/g. Thus, the value of *η*_ICA_ for ethanol-grown strain* Y. lipolytica *VKM Y-2373 (0.278 g/g) was comparable with that obtained with the best mutant strain* Y. lipolytica*, grown on rapeseed oil (0.250 g/g).

## 4. Conclusion

Thus, it can be concluded that both fermentation conditions such as pH and aeration and the composition of medium (concentration of zinc, iron, and itaconic acid (inhibitor of ICL–the key enzyme of ICA metabolism)) are effective factors controlling the ICA synthesis in* Y. lipolytica* yeast, grown on ethanol. The cultivation of* Y. lipolytica* VKM Y-2373 on a nutrient medium containing an increased content of zinc (0.6 mg/L), iron (1.2 mg/L), and 30 mM itaconic acid under selected optimal conditions at 29°C and various pH of the medium and the oxygen concentration (pH 5 and pO_2_ 20–25% (of saturation) during the growth period and pH 6 and pO_2_ 50–55% (of saturation) during the acid formation) ensured direct ICA production (90.5 g/L; selectivity of 80%; the mass yield of ICA (*Y*_ICA_) 0.77 g/g; and the energy yield of ICA (*η*_ICA_) 0.278 g/g).

## Figures and Tables

**Table 1:** The effect of cultivation parameters on ICA production by *Y. lipolytica.*

Parameters	Biomass (g/L)	ICA (g/L)	CA (g/L)	ICA/CA ratio	*Y*_ICA_ (g/g)
pH^1^					
3	8.9 ± 1.4	13.75 ± 2.14	13.89 ± 0.74	1 : 1	0.13
4	10.2 ± 1.6	20.81 ± 3.24	23.11 ± 0.65	1 : 1.1	0.21
4.5	11.2 ± 1.7	37.12 ± 2.67	40.15 ± 1.93	1 : 1.1	0.35
5	10.8 ± 1.6	40.64 ± 1.92	37.90 ± 1.54	1.1 : 1	0.38
5.5	10.8 ± 0.3	50.55 ± 0.64	38.14 ± 4.05	1.3 : 1	0.47
6	11.0 ± 0.3	59.00 ± 3.52	30.60 ± 6.77	1.9 : 1	0.55
6.5	10.1 ± 0.2	48.50 ± 3.54	28.20 ± 0	1.7 : 1	0.48
7	8.1 ± 1.3	12.33 ± 1.98	12.10 ± 1.89	1 : 1	0.17

Oxygen concentration (% from saturation)^2^					
5–10	7.6 ± 1.2	4.3 ± 0.6	4.1 ± 0.42	1 : 1	0.10
20–25	10.3 ± 1.6	35 ± 5.44	21 ± 3.27	1.7 : 1	0.33
30–35	10.7 ± 1.7	53.2 ± 8.27	28 ± 1.53	1.9 : 1	0.50
40–45	11.2 ± 1.8	53.68 ± 8.36	27 ± 1.37	2 : 1	0.52
50–55	11.0 ± 0.3	59.0 ± 3.52	29.0 ± 0.27	2 : 1	0.55
60–65	10.4 ± 1.6	46.5 ± 7.23	24.5 ± 0.43	1.9 : 1	0.53
75–80	9.0 ± 1.4	35.3 ± 5.49	19.6 ± 1.63	1.8 : 1	0.45

^1^The experiments were performed at 29°C and dissolved oxygen concentration (pO_2_) 50% (of saturation); ^2^the experiments were performed at 29°C and pH 6; values are mean ± standard deviation (*n* = 2).

**Table 2:** The effect of itaconic acid on ICA production.

Itaconic acid (мМ)	Biomass (g/L)	ICA (g/L)	CA (g/L)	ICA/CA ratio	*Y*_ICA_ (g/g)
0	10.30 ± 1.60	64.30 ± 7.18	23.72 ± 0.58	2.7 : 1	0.60
7.5	9.34 ± 0.91	61.78 ± 14.46	23.16 ± 0.84	2.7 : 1	0.65
20	9.50 ± 1.47	69.61 ± 7.63	17.50 ± 0.10	4.0 : 1	0.77
30	9.00 ± 0.01	72.19 ± 2.73	17.60 ± 0.71	4.1 : 1	0.77
40	9.00 ± 0.64	68.00 ± 3.51	17.00 ± 1.23	4 : 1	0.68

Values are mean ± standard deviation (*n* = 2).

**Table 3:** Comparative data on the processes of ICA production with the use of *Y. lipolytica*.

Strain	Substrate	ICA (g/L)	CA (g/L)	ICA/CA ratio	Productivity (g/l·h)	*Y*_ICA_ (g/g)	References
*Y. lipolytica* NMM-149	*n*-Alkanes	85.0	16	5.3 : 1	n.d.	1.23	[17]
*Y. lipolytica *704	Ethanol	66.0	31	2.1 : 1	n.d.	0.66	[23]
*Y. lipolytica* EH59	Sunflower oil	93.0	82.3	1.1 : 1	n.d.	0.65	[16]
*Y. lipolytica* VKM Y-2373	Rapeseed oil	70.6	22.4	3.2 : 1	0.797	0.82	[19]
*Y. lipolytica* 704-UV4-A/NG50	Rapeseed oil	86.0	20.0	4.3 : 1	0.970	0.95	[19]
*Y. lipolytica *UV/NG	Rapeseed oil	88.7	15.1	6 : 1	n.d.	0.90	[21]
*Y. lipolytica* VKM Y-2373	Ethanol	90.5	22.6	4 : 1	1.15	0.770	The present study
